# Which Company Characteristics Make a Food Business at Risk for Food Fraud?

**DOI:** 10.3390/foods10040842

**Published:** 2021-04-13

**Authors:** Saskia M. van Ruth, Onno Nillesen

**Affiliations:** 1 Food Quality and Design Group, Wageningen University and Research, P.O. Box 17, 6700 AA Wageningen, The Netherlands; 2 School of Biological Sciences, Queen’s University Belfast, 19 Chlorine Gardens, Belfast, Northern Ireland BT9 5DL, UK; 3 PwC, Thomas R. Malthusstraat 5, 1066 JR Amsterdam, The Netherlands; onno.nillesen@planned.nu; 4 PlanNed, Stephensonweg 14, 4207 HB Gorinchem, The Netherlands

**Keywords:** food adulteration, food crime, food fraud assessment, fraud risk, fraudster, fraud vulnerability, offence

## Abstract

Fraud can happen to any food business, but some sectors show more historical evidence of food fraud than others. This may be due to particular company characteristics that affect a company’s level of vulnerability. In the current study, we examined the relevance of the industry segment, business size, and location of food businesses on their food fraud vulnerabilities. Over 8000 food fraud vulnerability self-assessments conducted by food businesses active in 20 industry segments located in five continents were collected and the data analyzed. Results revealed that a company’s industry segment (chain and tier) affects its fraud vulnerability greatly and to a larger extent than the size of the business. The effect of industry segment on fraud vulnerability appears fairly similar across continents, whereas the effect of business size exhibits large geographical variation. The results demonstrate that those involved in animal product supply chains and end of chain nodes (catering, retail) are most vulnerable, and so are larger businesses, and businesses located in Africa and Asia. Current results imply that company characteristics are important determinants of the level of fraud vulnerability, and they may be used reversely in the future, i.e., as predictors of vulnerability.

## 1. Introduction

Food supply chains have become increasingly globalized [1] and so has food fraud. Food fraud is intentional deceit for economic gain with adverse human activity at the core of the problem [2]. Some of the businesses involved are offenders themselves and deliberately defraud their customers. Others are victimized by fraudsters earlier in the chain and pass on the illicit products to their own customers unknowingly [3]. 

**Figure 1:**
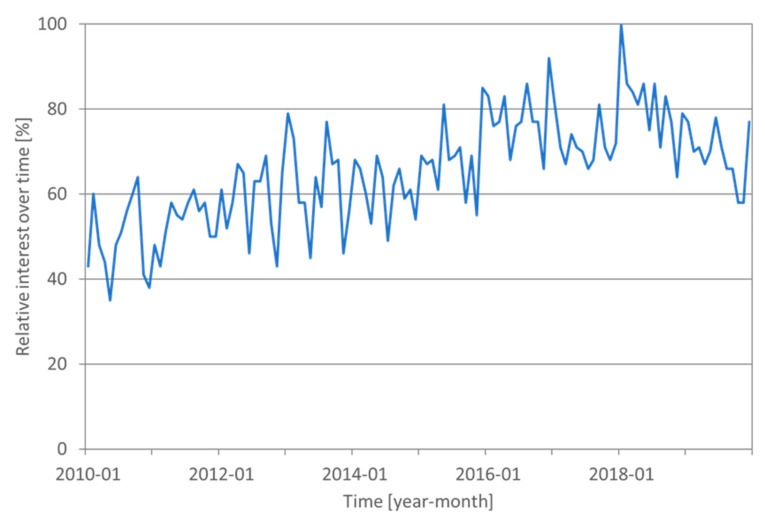
Evolvement of keyword search frequency in Google for “food fraud” in the period 2010–2020 (worldwide).

Inter- and intra-organizational food fraud can have major implications for supply chain performance. High-profile food fraud scandals result in substantial damage to the reputations of retailers, food manufacturers, and entire food industry segments. Moreover, these illegal activities have significant and long-lasting social consequences: they may pose health risks and they erode consumer confidence. In this context, the melamine milk scandal in China that took place in 2009 and resulted in hundred-thousands of hospitalized infants is an atrocious example, with consumer confidence in domestic infant formula remaining low, even after a decade [4]. Another unfavorable highlight was the EU horsemeat scandal in 2013. Due to the various food fraud incidents, food fraud gradually received more attention, which is reflected, for instance, in the frequency of the search term “food fraud” in Google (Figure 1). It also resulted in the food industry eventually recognizing that food fraud is seriously spiraling out of control and requires attention [5].

To design appropriate interventions in this area, it is crucial to comprehend the ecology of food fraud. Because it all revolves around illicit human behavior, a criminological approach is the most obvious. One of the well-established criminological theories for that matter is the Routine Activities Theory which uses three elements to explain crimes: the motivated offender, the suitable target, and lack of guardianship [6]. The first two are considered to entail the threats, and the last element (lack of) potential counteracting measures. From it follows that creation of an environment that is less vulnerable and less hospitable will inhibit fraudsters or at least discourage offender activity. Using this conceptual model and with consideration of evidence from natural and social sciences, 50 factors which determine food businesses’ vulnerability to food fraud have been identified [7]. They differ from food safety risk factors as socio-economic factors and criminal behavior need to be considered too [8]. These factors have been implemented into a practical fraud vulnerability assessment for food supply chain actors [7]. This assessment was transformed into an application which was available on the Apple and Google Play stores between 2016 and 2019, free of charge. The assessment has been widely used in food supply chains and also for scientific comprehension of the fraud vulnerabilities in milk [9,10], olive oil [11], and spice supply chains [12]. However, it is unknown which, and to what extent, company characteristics are determinants for the susceptibility to food fraud. In addition to this particular type of assessment, others have been developed. An overview of tools and their adoption in the food industry has been reported. They include the United States Pharmacopeia Food Fraud Mitigation tool, the Food Fraud initial Screening Tool, and the Campden Threat and Critical Control Points tool, among others [8]. 

Certain food types, geographical sources, associated supply chains [13] and business characteristics [14,15,16] are seen as having historically higher levels of concern with regard to food fraud. In the current study, we try to comprehend these vulnerabilities by exploring particular characteristics of businesses, such as the industry segment they are active in, their business size, and their location, that may influence their levels of vulnerability to fraud across over 8000 businesses active in global food supply chains.

## 2. Materials and Methods

### 2.1. Food Fraud Vulnerability Assessments 

Food fraud vulnerability assessments (FFVA) identify weaknesses or flaws that create opportunities for food fraud. The FFVA employed in this study consisted of 50 questions and associated three level answering grids (low-, medium-, and high vulnerability). Each question relates to identified, science-based fraud factors (indicators): nine for opportunities, 20 for motivations, and 21 for control measures [7]. The questions are listed in Appendix A. The full tool can be accessed free of charge through the Safe Supply of Affordable Food for Everyone Everywhere (SSAFE) website (www.ssafe-food.org, accessed on 1 March 2021). The assessment was developed and tested through a very extensive, interactive, and iterative procedure with representatives from businesses in food supply chains, authorities, and the scientific community from around the globe [7]. A free online version of the FFVA was developed by the SSAFE in partnership with Wageningen University, Free University of Amsterdam, and food industry leaders around the world. It aims to help companies to map their vulnerabilities and to strengthen their controls. An online application was subsequently developed by SSAFE and PricewaterhouseCoopers (PwC) which was made available free of charge to businesses in food supply chains. Although the spreadsheet version of the FFVA with all questions and answering options is still freely downloadable at the SSAFE website, the SSAFE/PwC online application is currently no longer available.

### 2.2. Correspondents: Assessed Businesses

**Figure 2:**
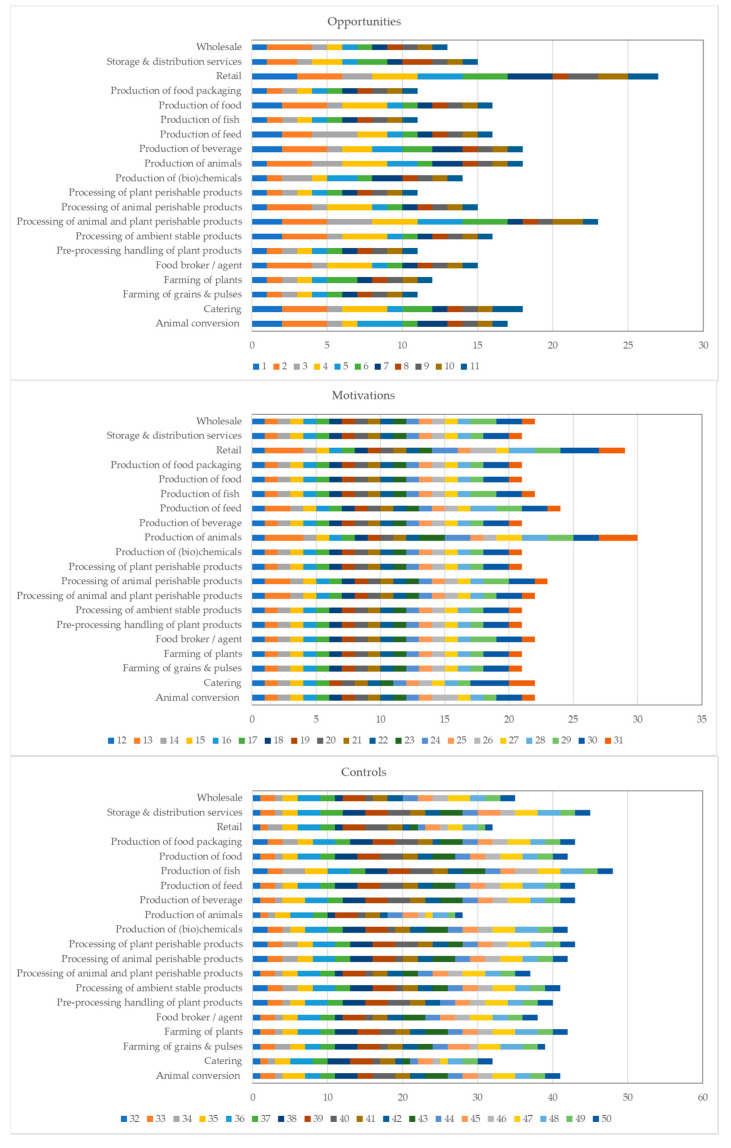
Overview of modes of the scores for individual questions of the food fraud vulnerability assessment per industry segment collated for opportunities, motivations and controls (global data, *n* = 8021). Numbers refer to fraud factor/question numbers, which are listed in Table 1. Animal conversion (*n* = 26); Catering (*n* = 80); Farming of grains and pulses (*n* = 49); Farming of plants (*n* = 279); Food broker/Agent (*n* = 163); Pre-processing handling of plant products (*n* = 210); Processing of ambient stable products (*n* = 757); Processing of animal perishable products (*n* = 363); Processing of animal and plant perishable products (*n* = 107); Processing of plant perishable products (*n* = 286); Production of (bio) chemicals (*n* = 170); Production of animals (*n* = 23); Production of beverage (*n* = 476); Production of feed (*n* = 66); Production of fish (*n* = 113); Production of food (*n* = 3494); Production of food packaging (*n* = 874); Retail (*n* = 47); Storage and distribution services (*n* = 346); and Wholesale (*n* = 92). A score of 1 indicates low vulnerability, 2 medium vulnerability, and 3 high vulnerability for the opportunities and motivations. For controls, the order is reversed.

Food fraud vulnerability was self-assessed voluntarily by 8021 businesses involved in food supply chains world-wide using the SSAFE FFVA described in the previous section. Four hundred fifty-two were located in Africa, 4361 in North and South America, 1341 in Asia, 1458 in Europe, and 409 in Oceania. They covered a range of 20 food industry segments, i.e., commodity supply chains and supply chain tiers (listed in the legend of Figure 2). Businesses were, for instance, involved in farming of plants or animals, pre-processing handling, food processing, wholesale, retail, and catering, as well as the production of packaging materials and feed. Businesses were allocated to the following business size groups: Nil–100, 101–500, 501–1000, 1001–10,000, and 10,000 or more employees. Companies filled out the FFVA to self-assess their organization and supply chain in the free online application for themselves, to obtain the FFVA for their organization. FFVA data were collected while anonymity of the businesses was ensured. Only identity-related information (meta-data) in regard to industry segment, business size at category scale, and location at continent level was retrieved and only if voluntarily provided by the businesses. Data were collected over the period 2016–2019.

### 2.3. Data Processing and Analysis

The distribution of the companies across industry segments was compared for the five continents to ensure that balance existed and that bias due to imbalance was avoided. The proximity matrix based on Pearson correlation coefficients was established.

The answer choices selected by the businesses in the SSAFE FFVA were transformed into a scoring system with assignment of a score of 1, 2, or 3 to the subsequent answering options. This meant that a high vulnerability option for the opportunities and motivations related questions was assigned a score of 3 and the low vulnerability option a score of 1, whereas for the controls related questions the order was reversed. The data resulted in a data matrix of 8021 businesses × 50 questions and, hence, 40,050 data points. 

Because the assessments resulted in ordinal data, i.e., the variables have natural, ordered categories and the distances between the categories are not known, a nonparametric approach was selected for the evaluation and interpretation of the data. The relative frequencies of the answer choices were calculated for each question across industry segments and across continents, and modes (most frequently selected options) were determined. Modes were also calculated across food fraud categories for comparison of continents. Significance of the effect of industry segment and business size was determined for each fraud factor and for each continent individually in a multifactor analysis using nonparametric tests (two-tailed Kruskal-Wallis test, fraud factor × industry segment and fraud factor × business size). XLstat (Addinsoft, New York, NY, USA) was used for all statistical analyses above. A significance level of *p* < 0.05 was applied throughout the study.

## 3. Results and Discussion

### 3.1. General Evaluation of the Characteristics Determining Fraud Vulnerability Levels

Food fraud self-assessments were conducted by 8021 companies across five continents which were active in a wide range of food industry segments—from farming of plants to production of beverages and from food packaging production to retail (Figure 2). A very similar distribution was observed across the five continents. Relative distribution across the industry segments was highly correlated for the five continents with correlation coefficients varying from 0.931 for the correlations of Africa and the other continents up to 0.988 for Europe and the other continents. Therefore, little bias resulting from interactions between continent and industry segment is expected. The modes of the answer options selected by the businesses are presented for each fraud factor and food industry segment in Figure 2. They are divided graphically into the three key elements: opportunities, motivations, and controls. For opportunities the low vulnerability option (value = 1) was selected most frequently across the board, i.e., 74% of modes across questions and food industry segments are associated with low vulnerability. Both the medium and the high vulnerability answer options were associated with the modes of 13% of the opportunities-related fraud factor questions. For the motivations-related fraud factors, low vulnerability was selected most frequently (89% of the modes was associated with the low vulnerability option), followed by the medium vulnerability option (10%). Hence, motivations scored somewhat lower in terms of fraud vulnerability contribution than the opportunities. For controls, the most common mode for the control-related fraud factors is the medium vulnerability option (56%). It is followed by the low vulnerability option (28%). Overall, this means that the vulnerability to food fraud is often perceived as relatively low when it comes to motivations and somewhat higher in regard to opportunities, with lack of adequate controls being considered to contribute to a larger extent to fraud vulnerability. These results are in agreement with small scale assessments which compared fraud vulnerability across the fish, meat, milk, olive oil, organic banana, and spice industries in Europe [15]. These overall profiles provide a first, general impression of the large dataset. However, more detailed information is available which allows examination of the impact of food industry segment, business size, and businesses’ geographical location, which are described in the following sections.

### 3.2. The Industry Segment

#### 3.2.1. Differences across Segments

The assessments showed that food industry segments differed in their level of vulnerability for the three key elements (Figure 2). At first sight, level of opportunities for food fraud across the supply chains as reported by the businesses varied considerably across segments with especially businesses involved in the processing of plant and animal perishable products, and the retail sector noting higher vulnerability.

Less variation across segments is observed for motivations with the exception of businesses involved in the production of animals and retail presenting a slightly higher vulnerability. Regarding controls, businesses in animal production and upper tiers across supply chains (retail, catering) indicated fewer adequate controls (lower control scores = higher vulnerability).

After ranking of the industry segments according to threats (combined opportunities and motivations-related responses) based on the multifactor analysis results, it is striking that in particular the animal product supply chain appears to face more threats than businesses in other supply chains (Table 1). Animal conversion, production of animals, processing of animal and plant perishable products, processing of animal perishable products rank among the top five. Those reporting fewest threats are those businesses involved in the first steps of the plant product supply chains (farming of plants and preprocessing handling of plant products), storage, distribution and wholesale, and food packaging production. Because threats may be mitigated by implemented control measures, the level of adequate controls or the lack of controls is also of interest. For this aspect, mostly businesses in the first and final steps of the supply chains rank high, which means they are lacking adequate fraud controls. On the one hand, these are businesses involved in farming of plants, farming of grains and pulses, production of animals or those involved in animal conversion. On the other hand, it concerns end of chain actors, such as those involved in retail and catering. Businesses that hardly interact with food, such as wholesalers and food brokers/agents, also report lower levels of control measures. At the other end of the scale, i.e., those that report the most adequate controls, we find businesses in food/beverage/feed production, food packaging, and fish production. Those industry segments that face threats and have limited controls in place are obviously the most vulnerable to food fraud. These involve businesses in animal product supply chains as well as those at the end of the supply chain (retail, catering). Primary production businesses are far less vulnerable to fraud. These results indicate that the industry segment is an important determinant of food fraud vulnerability and has two dimensions. Both the type of commodity chain (animal more vulnerable than plant) and the tier in the chain (upper tiers more vulnerable than lower tiers) determine food fraud vulnerability.

Interestingly, these vulnerabilities align also with recorded food fraud incidents. For instance, animal products such as meat and edible offal, milk and milk products, fish and other seafood, and honey and royal jelly most fraud records most frequently noted for the period 2008–2013 in the USP database, the FoodShield database, and the EU Rapid Alert System for Food and Feed [17]. Similarly, products of animal origin are over-represented in an analysis of local Finnish food fraud cases [18].

Regarding the tier in the chains, e.g., primary production versus processing versus retail/catering, it has been shown that each tier faces a competitive environment with its own distinct characteristics and pressure. This was illustrated in a study on fraud vulnerability in the Netherlands which indicated the enhanced vulnerability of processors of liquid milk in comparison to their supplying farmers [19]. Similarly, the study of Guntburger et al. [20] indicated that Canadian primary producers felt relatively safe from food fraud in the past, whereas a higher rate of processors that expected to have been victimized in the past was noted, and an even higher rate for distributors (retailers) [20]. In a former study, food service operators (catering) also rated fraud vulnerability considerably higher than other tiers in food supply chains with particularly more opportunities and fewer controls noted [3]. This group indicated that they lacked overview and face limited span of control of their supplies along the supply chains. Without sufficient controls, they are more likely to be victimized by suppliers or by businesses in earlier steps in the supply chain. Especially complex supply chains may create blind spots [21]. On the other hand, the number of supply chains that are long, interconnected, multi-nodal, and complex in nature is ever increasing due to globalization [1]. 

Another important aspect along supply chains is the difference in level of information that exists between business entities. This information asymmetry is more likely to urge opportunistic behavior which in turn weakens the vulnerability of those towards the end of the chain in longer and more complex supply chains [22]. Interaction of informed expert sellers in one business entity which interact with uninformed customers in other entities down the food supply chains increases the vulnerability of the latter substantially. This potential for fraudulent behavior by expert suppliers with superior information is also seen in health care, legal and financial services, as well as in automobile and computer repair services [23].

It is known that culturally embedded behaviors resulting in unethical practices may become normalized across food chains. It may also have a contagious effect as businesses that may be disadvantaged through such practices are forced to use similar practices for survival. As a result, eventually, some segments/supply chains will become more contaminated than others [24].

#### 3.2.2. Variation in the Segment Effect across Continents

In order to understand the impact on individual factors in greater detail, the significance of the effect of industry segment on fraud vulnerability was determined separately for each continent for each of the 50 fraud factors (Table 2). Fraud vulnerability differed significantly across industry segments for 80% of these fraud factors (Table 2). Especially vulnerability resulting from fraud factors related to technical opportunities (97%) and opportunities in time and place (100%) were very much affected by the industry segment, and this holds for all continents. This means that considerable variation in terms of low and high vulnerability resulting from different levels of opportunities is present across the industry segments—anywhere in the world. For the motivations-related responses, 74% of the factors related to economic drivers and 71% of those related to cultural and behavioral drivers, which presented a significant difference across industry segments. Hence, industry segments differ significantly in the economic drivers they are facing, such as the supply and pricing of materials. This effect also varied more across continents: more variation in motivational drivers across segments was noted for businesses located in Africa, Asia, and Europe, and less for those based in the Americas and Oceania. Technical and managerial controls varied at an intermediate level across industry segments, with 58% and 75% of the factors across continents presenting a significant effect, respectively. However, the effects varied greatly across continents. They ranged from 9% of the technical controls factors showing significant differences across industry segments for Oceania up to 82% for Asia and Europe. Although Oceania showed also a lower number of fraud factors related to managerial controls significantly affected by industry segment (38%), the other continents exhibited more similar significance values (75–100% of the fraud factors presenting a significant effect of industry segment). To conclude, Africa, Asia, and Europe presented large differences in fraud vulnerability across industry segments, Americas to a lesser extent, and Oceania the least. Clearly, the industry segment is a key determinant of opportunities, motivations, and level of controls for food fraud around the globe.

### 3.3. The Business Size

#### 3.3.1. Differences across Business Size Groups

The size of a business affected the reported threats (i.e., opportunities and motivations) they are facing. According to Kruskal-Wallis tests, in general, businesses with fewer employees are confronted with significantly fewer threats than larger sized businesses. The larger ones perceived themselves as exposed to a higher level of opportunities and motivational drivers across their supply chains. On average, the level of control measures decreased slightly with increase in business size but the levels varied considerably. Although there are no reports linking food fraud to business size to the best of our knowledge, studies on economic fraud and business size relationships exist and some indicated the relevance of business size [25]. Generally, larger sized businesses usually suffer larger losses from this kind of fraud [26]. They are probably better known and more visible, and this kind of food businesses also interact with more suppliers, which increases their exposure. Secondly, larger businesses usually deal with larger orders and bigger sums of money, which may increase attractivity [27]. Thirdly, large businesses often do business across national borders [28]. Finally, large businesses usually have more complicated organizational structures that may favor internal threats [28]. 

#### 3.3.2. Variation in the Business Size Effect across Continents

The effect of business size was examined in the same manner as the effect of the industry segment described in the previous sections. Reported fraud vulnerability resulting from 49% of the fraud factors was significantly affected by the size of businesses (Table 2). This proportion is considerably lower than noted for the effect of the industry segment (80%) and this is observed for all six food fraud factor categories. Forty percent of the factors in the technical opportunities category and 30% of the factors in the opportunities in time and place category displayed significant differences between food business size groups. Motivational drivers were affected more because 54% of the factors related to economic drivers and 57% of those related to the cultural and behavioral drivers showed a significant different level of fraud vulnerability across businesses of different sizes. Fraud vulnerability resulting from presence or lack of adequate controls was influenced by business size to a similar extent as the motivational drivers: 58% and 55% of the fraud factors related to technical controls and managerial controls, respectively, exhibited significant differences. 

The effect of business size on fraud vulnerability varied considerably across continents and to a larger extent than the effect of industry segment. Oceania demonstrated the smallest effect with the fraud vulnerability of 22% of the fraud factors differing significantly across business size groups. On the contrary, Asia presented the largest effect of business size, i.e., for 87% of the fraud factors. Europe (37%), Americas (47%), and Africa (52%) presented intermediate effects. In conclusion, generally the impact of business size on fraud vulnerability is lower than the impact of industry segment, but it varies more with geography.

### 3.4. The Location

**Figure 3:**
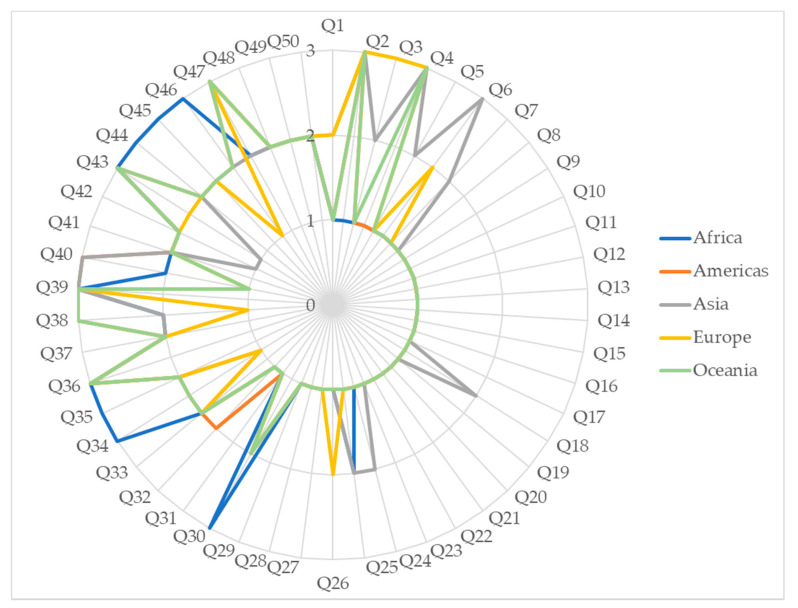
Spiderweb diagram of the modes of scores for each food fraud vulnerability assessment question (Q1, Q2, etc.) for businesses located in the five different continents. If modes of continents overlap, only one color is shown. A score of 1 on the axis indicates low vulnerability, 2 medium vulnerability, and 3 high vulnerability for the opportunities and motivations (Q1–Q31). For controls (Q32–50), the order is reversed.

For a first general impression of the impact of geography, the modes of all questions per continent were calculated. They are presented in a spider web diagram in Figure 3. The scores for the food fraud categories across continents agreed fairly well in terms of ranking: For instance, highest vulnerability scores are observed for technical opportunities and the two controls’ categories for all continents. There are, on the other hand, also geographical influences. The five continents were ranked according to the reported threats (scores for opportunities and motivations based on the multifactor analysis results), which resulted in the following order: Africa > Asia > Europe > Oceania > Americas. The levels of controls varied less on average, only the Americas showed a significant higher level of adequate controls compared to the other continents. The differences observed between geographical locations are in agreement with those of former studies which indicated geographical variation in fraud threats in regard to food commodities but also in many other areas [29].

### 3.5. Considerations Regarding the Determinants Industry Segment, Business Size, and Location

**Figure 4:**
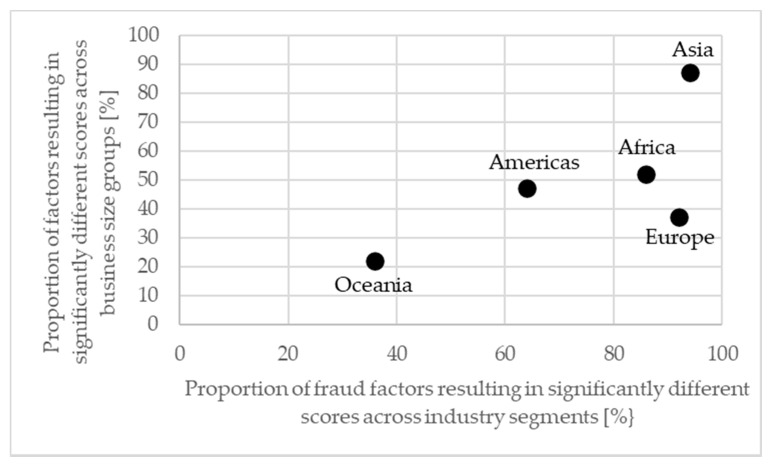
Comparison of the effects on variation in food fraud vulnerability responses of businesses in global food supply chains.

A final step in this study is the comparison of the effects of industry segment, business size, and geographical location of businesses in food supply chains on their fraud vulnerability. The proportion of fraud factors affected by either the industry segment or the business size is presented in Figure 4 for each continent. More food fraud factors exhibited significant differences due to industry segment (74% of the fraud factors on average) than due to business size (49%). The geographical component is more important for the effect of the business size than for the effect of the industry segment, which is indicated by the extent of the spread along the horizontal and vertical axis of the continents in Figure 4. Businesses in Oceania show the most uniform food fraud vulnerability with limited differences due to industry segment or business size, whereas at the other end of the scale we find Asia, where businesses differ substantially in their food fraud vulnerability patterns both in regard to industry segment and business size. Businesses in Africa, the Americas, and Europe show a similar intermediate effect of the size of businesses, but businesses located in the Americas show less effect of the industry segment than African and European businesses. It is obvious that the effect of the geographical location interacts with the effects of the other two company characteristics and needs to be considered when evaluating food fraud risks. 

### 3.6. Considerations Regarding the Study

The SSAFE tool was designed to cover a very broad scope and aimed to be used in practice by “any food business in the world”. As a consequence, the 50 questions cover a wide but still selected range of topics, and this leads to a rough measurement of the key elements. The SSAFE tool was developed in a Western context, although input from businesses around the globe was encouraged during the long trajectory of the development of the tool with, e.g., workshops in Singapore. Nevertheless, economic and cultural practices in Asia generally differ from those in the EU or the Americas. These backgrounds may affect the selection of situations that mimic the own situation best, as required with the SSAFE tool, especially when it concerns more sensitive topics. For instance, questions regarding the ethical business culture and corruption level may result, in general, in more socially desirable answers, but if this desirability bias has a cultural component, it may affect results at the continental level. This was one of the reasons to look also into the results per continent.

Finally, there is, at this stage, limited work showing the correlation between food fraud vulnerability and actual fraud prevalence in food supply networks. To the best of our knowledge, there is only one study. This study deals with fraud vulnerability and prevalence in Chinese milk supply chains [15]. More extensive studies to correlate vulnerabilities and prevalence would certainly enhance fine-tuning of these types of assessments and their predictive and preventative capabilities.

## 4. Conclusions

A company’s industry segment is a key determinant of its fraud vulnerability and is more important than the size of a business. The effect of the industry segment on fraud vulnerability interacts to some extent with its location, but the influence of the business size varies much more with the company’s location. These business’s characteristics affect fraud vulnerability in different ways by influencing the level of opportunities, motivational drivers, controls, or a mix of them. The industry segment affects and determines predominantly opportunities and to a lesser extent motivational drivers and availability of adequate controls. Business size affects the level of motivational drivers and controls rather than opportunities. Opportunities and motivations vary also with geographical location. From the companies assessed, those involved in animal product supply chains and end of chain tiers (catering, retail) were most vulnerable, and so were larger sized businesses, and businesses located in Africa and Asia. 

Certain businesses and employees thereof are constantly identifying new and ingenious ways to defraud their customers. For this reason, it is important for those with a stake in a company to devise ways of identifying and preventing fraud to protect their business interests. Comprehension of key characteristics of businesses leading to high fraud vulnerability and particular elements of fraud vulnerability assist decision making and help to design efficient interventions to combat food fraud.

## Figures and Tables

**Table 1:** Vulnerability ranking of industry segments resulting from threat level (scores for opportunities and motivations-related factors) and lack of adequate controls (scores for controls-related factors) from the multifactor analyses: 1 = highest vulnerability; 20 is lowest vulnerability.

Industry Segment	Opportunities + Motivations (Threats)	Controls
Animal Conversion	1	3
Production of Animals	2.5	1
Retail	2.5	4
Processing of Animal and Plant Perishable Products (mixed products)	4	10
Processing of Animal Perishable Products	5	17
Processing of Ambient Stable Products	6.5	13
Production of Feed	6.5	14
Production of Beverage	8	18
Production of Food	9	15.5
Production of (bio) Chemicals	10	12
Catering	11	2
Farming of Grains & Pulses	12	6
Food Broker/Agent	13	8
Processing of Plant Perishable Products	14	11
Production of Fish	15	20
Preprocessing handling of plant product	16	15.5
Production of Food Packaging	17	19
Farming of Plants	18.5	5
Storage & Distribution Services	18.5	9
Wholesale	20	7

**Table 2:** Significance of differences in fraud vulnerability scores for individual fraud factors due to industry segment (A) and business size (B) for food businesses located in five continents (Kruskal-Wallis test, two-tailed).

				A. Differences Due to Industry Segment					B. Differences Due to Business Size				
Key Element	Fraud Factor Category	Question nr	Fraud factor	Africa (*n* = 452)	Americas (*n* = 4361)	Asia (*n* = 1341)	Europe (*n* = 1458)	Oceania (*n* = 409)	Africa (*n* = 452)	Americas (*n* = 4361)	Asia (*n* = 1341)	Europe (*n* = 1458)	Oceania (*n* = 409)
Opportunities	Technical opportunities	1	Complexity of adulteration of raw materials	<0.0001	<0.0001	<0.0001	<0.0001	<0.0001	0.093	0.005	<0.0001	0.522	0.000
		2	Availability technology and knowledge to adulterate raw materials	<0.0001	<0.0001	<0.0001	<0.0001	0.003	0.05	0.026	0.162	0.989	0.093
		3	Fraud detectability in raw materials	0.018	<0.0001	0.000	<0.0001	<0.0001	0.098	<0.0001	0.078	0.321	0.061
		4	Availability technology and knowledge to adulterate final products	0.000	<0.0001	<0.0001	<0.0001	0.005	0.001	0.84	<0.0001	0.61	0.155
		5	Fraud detectability in final products	0.002	<0.0001	0.015	0.003	0.000	0.219	<0.0001	0.227	0.052	0.842
		6	Complexity of counterfeiting	0.000	<0.0001	<0.0001	<0.0001	0.301	0.022	0.153	<0.0001	0.662	0.17
		7	Detectability of counterfeiting	0.002	<0.0001	<0.0001	<0.0001	0.023	0.01	<0.0001	<0.0001	0.235	0.023
	Opportunities in time and place	8	Access to production lines/processing	<0.0001	<0.0001	<0.0001	<0.0001	0.004	0.276	0.572	0.006	0.15	0.804
		9	Transparency in chain network	0.000	<0.0001	<0.0001	0.003	0.000	0.004	0.075	<0.0001	0.281	0.09
		10	Historical evidence of fraud in raw materials	<0.0001	<0.0001	<0.0001	<0.0001	0.006	0.157	0.000	<0.0001	0.56	0.692
		11	Historical evidence of fraud in final products	0.013	<0.0001	<0.0001	<0.0001	0.004	0.178	0.343	<0.0001	0.663	0.186
Motivations	Economic drivers	12	Supply and pricing raw materials	0.03	0.008	<0.0001	<0.0001	0.423	0.43	<0.0001	0.001	0.196	0.848
		13	Valuable components or attributes	0.001	<0.0001	<0.0001	<0.0001	0.015	0.009	0.197	<0.0001	0.632	0.746
		14	Economic health own company	0.000	0.364	<0.0001	0.016	0.411	0.019	0.664	0.000	0.002	0.098
		20	Economic health supplier	0.001	0.517	0.126	0.002	0.129	0.018	0.006	0.011	0.019	0.006
		26	Economic health sector	0.009	0.007	<0.0001	<0.0001	0.141	0.016	0.216	<0.0001	0.205	0.455
		30	Level of competition branch of industry	0.001	<0.0001	0.014	<0.0001	0.426	0.007	0.115	0.002	0.184	0.088
		31	Prices asymmetries	0.001	0.025	<0.0001	<0.0001	0.377	0.009	0.002	<0.0001	0.407	0.689
	Cultural and behavioral drivers	15	Organizational strategy own company	0.004	0.152	0.002	0.107	0.034	0.004	0.813	0.036	0.647	0.059
		16	Ethical business culture own company	<0.0001	0.316	<0.0001	0.023	0.067	0.009	0.012	<0.0001	0.13	0.045
		17	Criminal offences own company	0.007	0.191	0.026	0.004	0.871	0.000	0.026	0.000	0.007	0.008
		18	Corruption level country own company	0.004	<0.0001	<0.0001	0.002	0.13	0.092	<0.0001	0.013	0.183	0.059
		19	Financial strains supplier	0.463	0.049	<0.0001	0.004	0.025	0.002	0.307	<0.0001	0.002	0.474
		21	Organizational strategy supplier	0.000	0.359	<0.0001	0.001	0.297	0.372	0.000	0.009	0.768	0.022
		22	Ethical business culture supplier	0.001	<0.0001	0.02	0.009	0.412	0.048	0.03	0.007	0.399	0.107
		23	Criminal offences supplier	0.003	0.021	<0.0001	0.058	0.054	0.002	0.063	<0.0001	0.008	0.089
		24	Victimization supplier	<0.0001	0.029	<0.0001	0.000	0.017	0.496	0.361	<0.0001	0.246	0.226
		25	Corruption level country supplier	0.002	0.000	0.02	0.002	0.13	0.05	<0.0001	0.005	0.001	0.004
		27	Criminal offences customer	0.034	0.326	<0.0001	0.001	0.96	0.14	0.457	<0.0001	0.073	<0.0001
		28	Ethical business culture branch of industry	0.037	0.277	<0.0001	<0.0001	0.003	0.201	0.72	<0.0001	0.086	0.028
		29	Historical evidence branch of industry	0.078	0.000	<0.0001	<0.0001	0.333	0.04	0.308	<0.0001	0.409	0.019
Controls	Technical controls	32	Fraud monitoring system raw materials	0.034	0.062	<0.0001	0.005	0.05	0.000	0.283	<0.0001	0.002	0.124
		33	Verification of fraud monitoring system raw materials	0.221	0.259	0.004	0.078	0.228	0.024	0.156	<0.0001	0.002	0.477
		34	Fraud monitoring system final products	0.285	0.014	0.000	0.003	0.946	0.002	0.493	<0.0001	0.204	0.106
		35	Verification of fraud monitoring system final products	0.48	0.061	0.002	0.000	0.799	0.119	0.068	<0.0001	0.102	0.244
		36	Information system own company	0.002	<0.0001	<0.0001	<0.0001	0.373	0.179	0.405	<0.0001	<0.0001	0.106
		37	Tracking and tracing system own company	0.000	0.143	0.149	<0.0001	0.251	0.001	0.000	0.017	0.122	0.112
		42	Fraud monitoring system supplier	<0.0001	0.003	<0.0001	0.033	0.265	0.043	0.003	<0.0001	0.042	0.398
		43	Information system supplier	0.003	0.002	0.001	0.127	0.367	0.009	<0.0001	<0.0001	0.3	0.108
		44	Tracking and tracing system supplier	<0.0001	0.058	<0.0001	<0.0001	0.105	0.000	0.000	0.001	0.002	0.279
		46	Fraud control guidance industry	0.019	0.17	0.055	0.006	0.4	0.001	0.025	0.016	0.006	0.953
		50	Contingency plan	0.001	0.272	0.007	0.000	0.048	0.002	0.116	0.011	0.029	0.381
	Managerial controls	38	Integrity screening own employees	0.005	0.01	0.000	0.002	0.11	0.375	0.01	<0.0001	0.000	0.506
		39	Ethical code of conduct own company	0.000	0.277	<0.0001	0.001	0.449	0.421	0.068	<0.0001	<0.0001	0.101
		40	Whistle blowing own company	0.001	0.018	<0.0001	0.031	0.007	0.091	0.01	<0.0001	<0.0001	0.016
		41	Contractual requirements supplier	0.001	0.018	<0.0001	<0.0001	0.038	0.191	0.509	<0.0001	<0.0001	0.945
		45	Social control chain network	0.008	0.09	0.016	<0.0001	0.044	0.015	0.013	0.018	0.018	0.295
		47	National food policy	0.003	0.019	<0.0001	<0.0001	0.444	0.001	0.071	0.697	0.003	0.033
		48	Law enforcement local chain	0.223	0.004	<0.0001	0.001	0.183	0.053	0.028	0.343	0.002	0.012
		49	Law enforcement chain network	0.141	0.018	<0.0001	0.037	0.117	0.237	0.14	0.413	0.017	0.627

## Data Availability

Not available.

## Appendix A. The Fifty Questions of the SSAFE Food Fraud Vulnerability Assessment Tool

**Nr**	**Question**
1	How simple or complex is adulteration of your raw materials?
2	How generally available is the technology and knowledge on adulteration of your raw materials?
3	How easily would adulteration of your raw materials be detected and with what kind of methods?
4	How generally available is the technology and knowledge allowing adulteration of your final products?
5	How easily would adulteration of your final products be detected and with what kind of methods?
6	How simple or complex is counterfeiting of your final product?
7	How easily would counterfeited final product be detected, and with what kind of methods?
8	How would you describe the production processing lines of your company?
9	How would you describe the chain network that you are part of?
10	How many fraud incidences of similar raw materials have been reported?
11	How many fraud incidences of similar final products have been reported?
12	How would you define supply and pricing of your raw materials?
13	Do special attributes or components determine the value of your raw materials?
14	How would you describe the economic conditions of your company?
15	What are the characteristics of the organizational/business strategy of your company?
16	How would you describe the ethical business culture in your company?
17	Has your company been involved in criminal offences previously?
18	How would you rate the corruption level (according to the Transparency International Corruption Perception Index) in the countries in which your company is active?
19	How would you describe the financial strains imposed by your own company on your direct supplier(s) ?
20	How would you describe the economic conditions of your direct supplier(s)?
21	What are the characteristics of the organizational/business strategy of your direct supplier(s) ?
22	How would you describe the ethical business culture of your direct supplier(s) ?
23	Has/have your direct supplier(s) been involved in criminal offences previously ?
24	Has/have your direct supplier(s) been victimized by food fraud committed by their suppliers, customers or other parties?
25	How would you rate the corruption level (according to the Transparency International Corruption Perception Index) in the countries in which your direct supplier(s) and customers are active?
26	How would you describe the economic conditions in your branch of industry?
27	Has/have your customer(s) been involved in criminal offences previously ?
28	How would you describe the ethical business culture in your branch of industry (i.e., other meat processing companies)?
29	How common are criminal offences in your branch of industry?
30	How would you rate the level of competition in your branch of industry?
31	How would you define price asymmetries created by the legal framework?
32	How would you describe the fraud related parts of your raw material monitoring control system of your company?
33	Are the fraud monitoring tasks of your raw material control system verified in your company?
34	How would you describe the fraud related parts of your final product monitoring control system of your company?
35	Are the fraud monitoring tasks of your final product control system verified in your company?
36	How extensive is the information system for internal control of mass balance flows in your company?
37	How extensive is the tracking & tracing system of your company?
38	Is integrity screening of employees common procedure in your company?
39	Is there an ethical code of conduct or guidelines in place and embedded in your company?
40	Is there a whistle blowing system (system for reporting assumed fraud practices) in place in your company?
41	What is characteristic for your contractual requirements with your direct suppliers?
42	What features the fraud control system of your direct supplier(s)? An alternative for this question is that direct suppliers fill out the tool themselves with respect to the control measures as defined for the company
43	How extensive is the information system for control of mass balance flows of your direct supplier(s)? This question can be only asked direct to the supplier(s)
44	How extensive is the tracking & tracing system of your direct supplier(s)? An alternative for this question is that direct supplier(s) fill out the tool themselves with respect to the T&T system as defined for the company
45	How would you describe the social control and transparency of actions in your supply chain network?
46	How well established is the fraud control support by your branch of industry (e.g., branch of oils and fats)
47	How would you describe your national food policy?
48	How well are fraud related laws enforced in your local supply chain?
49	How well are fraud related laws enforced in your international supply chain network?
50	Does your company have fraud contingency measures in place?
